# Supplementary material for: Inferring Bacterial Community Interactions and Functionalities Associated with Osteopenia and Osteoporosis in Taiwanese Postmenopausal Women
Source: Microorganisms. 2023 Jan 17;11(2):234. doi: 10.3390/microorganisms11020234 (PMC9959971; doi:10.3390/microorganisms11020234)
Supplement: Supplementary file 1 [file microorganisms-11-00234-s001.zip › microorganisms-2051370-supplementary.pdf]

Supplementary information

Inferring bacterial community interactions and functionalities associated with osteopenia and osteoporosis in Taiwanese postmenopausal women

Tables

Table S1. Characteristics of the participants

	Healthy subjects	Osteopenia	Osteoporosis	*p* value
Age	56.0 ± 8.5	57.7 ± 8.3	53.5 ± 8.9	0.352
Body mass index	22.6 ± 7.7	23.8 ± 2.8	21.2 ± 2.0	0.168
Years after menopause	11.9 ± 11.2	15.8 ± 9.9	19.8 ± 12.2	0.199
T-score	-0.5 ± 0.3	-1.8 ± 0.3	-3.5 ± 0.7	-

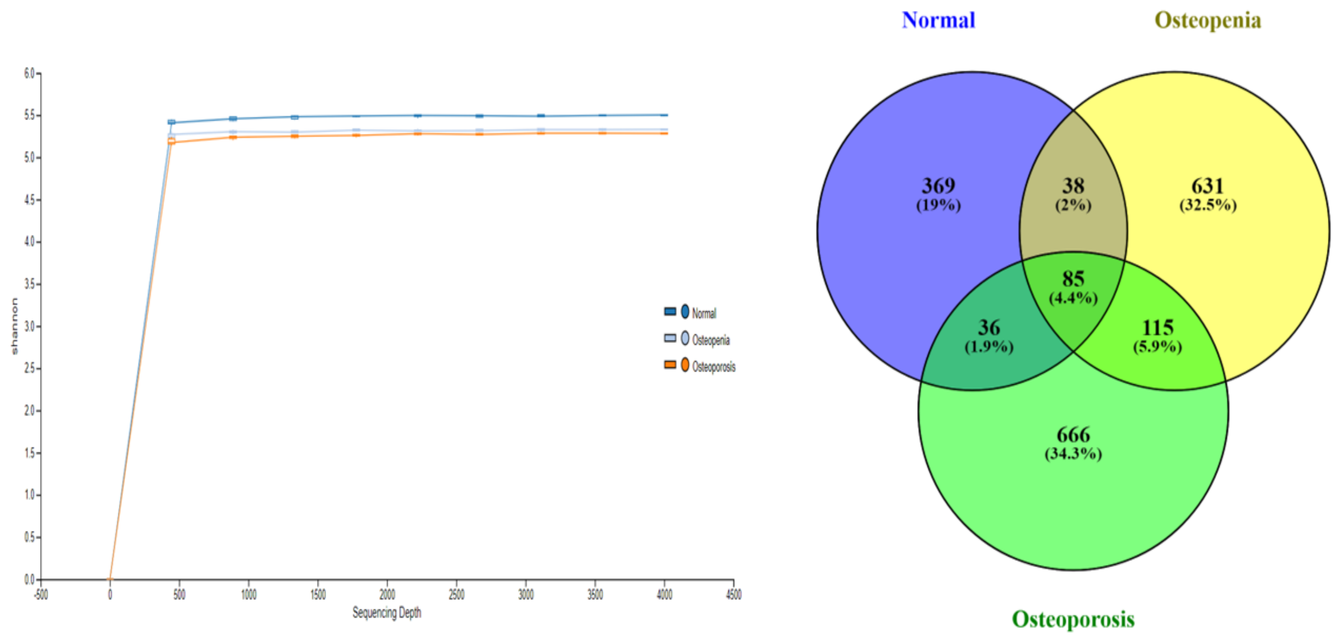

(A) (B)

Figure S1. Comparison of rarefaction (A) at lowest sequence depth among the experimental groups; normal control, osteopenia and osteoporosis. Venn diagram (B) representing the shared and unique ASVs among the three experimental groups

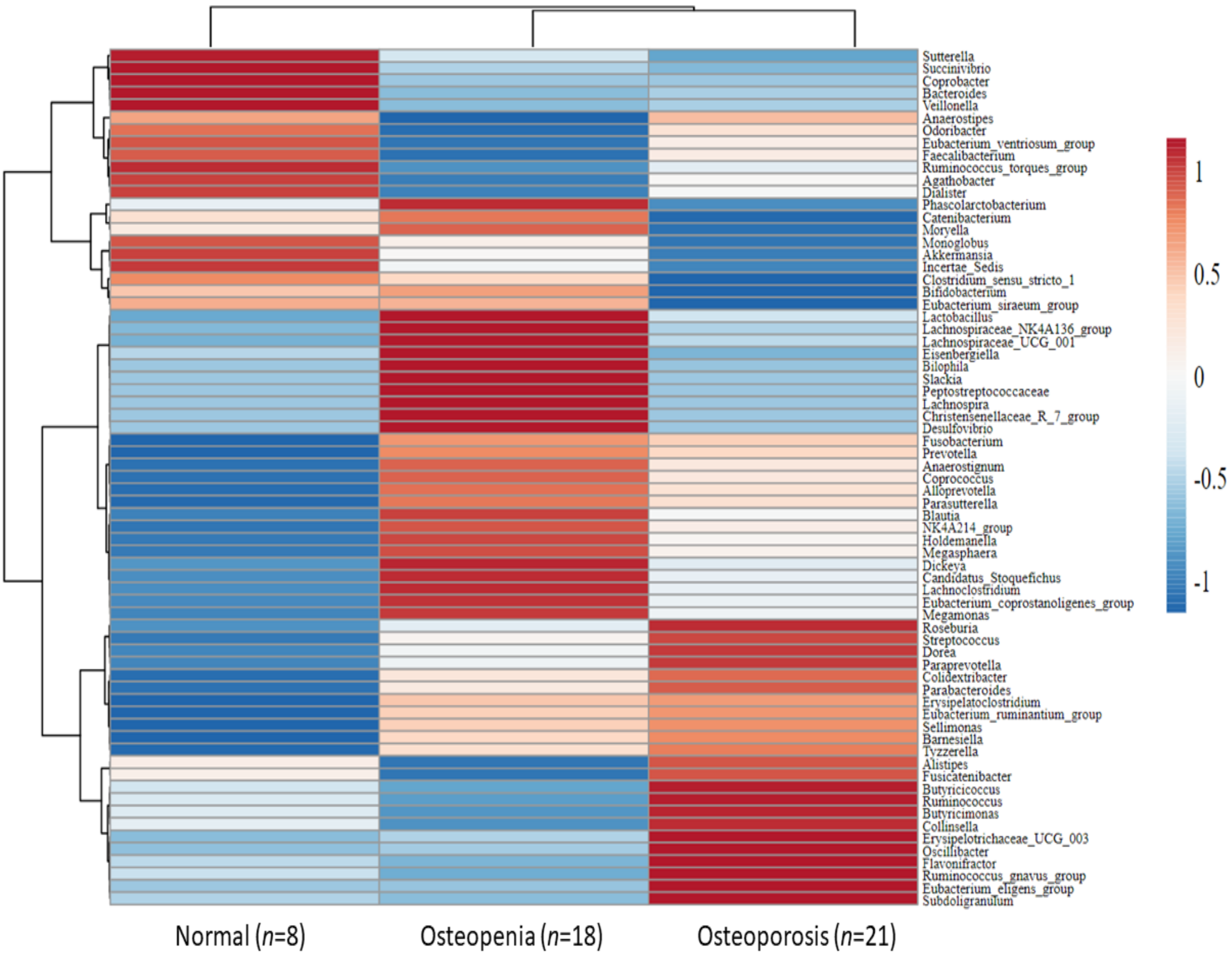

Figure S2. Heatmap indicating bacterial community abundance pattern at genus level among the three groups.

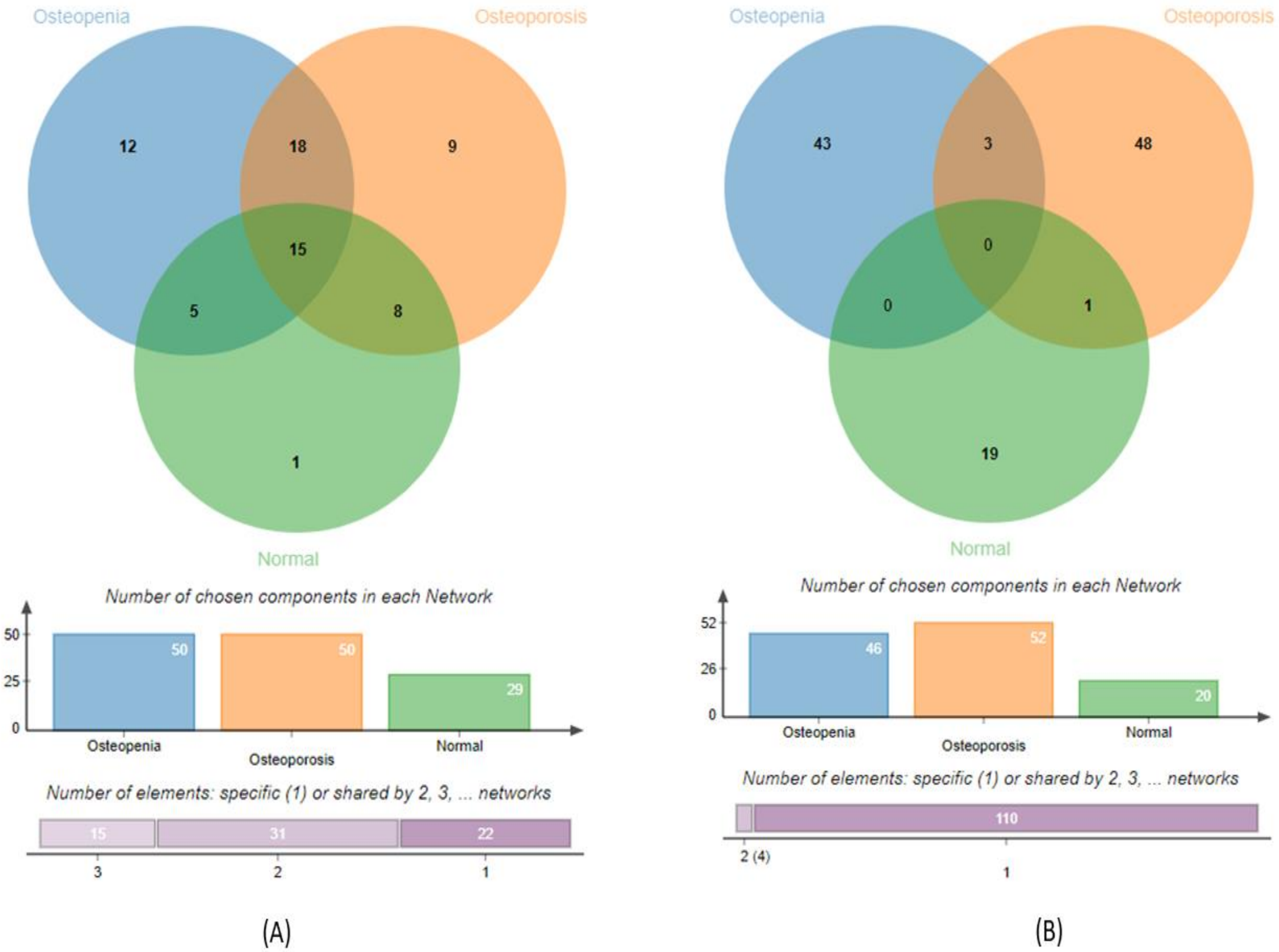

Figure S3. Network characterization of bacterial community co-occurrence network among the experimental groups. The Venn diagrams and bar plots represent network nodes (A) and edges (B) observed in healthy control, osteopenia, and osteoporosis in Taiwanese postmenopausal women.
